# Production and characterization of a new detergent-stable keratinase expressed by *Pedobacter* sp. 3.14.7, a novel Antarctic psychrotolerant keratin-degrading bacterium

**DOI:** 10.1186/s43141-022-00356-x

**Published:** 2022-05-25

**Authors:** P. Rios, B. Bezus, S. Cavalitto, I. Cavello

**Affiliations:** Centro de Investigación y Desarrollo en Fermentaciones Industriales, Facultad de Ciencias Exactas (CINDEFI, CCT La Plata-CONICET, UNLP), Universidad Nacional de la Plata, Calle 47 y 115, (B1900ASH), 1900 La Plata, Argentina

**Keywords:** Cold active keratinase, *Pedobacter*, Keratin biodegradation

## Abstract

**Background:**

Antarctica is one of the harshest environments in the world. Despite this fact, it has been colonized by microorganisms, which had to develop different adaptations in order to survive. By studying their enzymes, we can harness these adaptations in order to use them in various industrial processes. Keratinases (E.C. 3.4.99.11) are characterized by their robustness in withstanding extreme conditions and, along with other enzymes, are commonly added to laundry detergents, which makes their study of industrial interest.

**Results:**

In this work, a novel keratinase producer, *Pedobacter* sp. 3.14.7 (MF 347939.1), isolated from Antarctic birds’ nests, was identified. This psychrotolerant isolate displays a typical psychrotolerant growth pattern, with an optimal temperature of 20 °C (μmax=0.23 h^−1^). After 238 h, maximum proteolytic (22.00 ± 1.17 U ml^−1^) and keratinolytic (33.04 ± 1.09 U ml^−1^) activities were achieved with a feather sample conversion of approximately 85%. The keratinase present in crude extract was characterized as a metalloprotease with a molecular weight of 25 kDa, stable in a wide range of pH, with an optimum pH of 7.5. Optimum temperature was 55 °C. Wash performance at 20 °C using this crude extract could remove completely blood stain from cotton cloth.

**Conclusion:**

We report a new keratinolytic bacteria from maritime Antarctica. Among its biochemical characteristics, its stability in the presence of different detergents and bleaching agents and its wash performance showed promising results regarding its potential use as a laundry detergent additive.

## Background

It is well known that Antarctica is one of the most adverse regions in the planet, due to particular characteristics such as its cold weather, lack of moisture, strong winds, and inaccessibility [1]. Despite this, prokaryotes have managed to be the dominant biomass component of these ecosystems and play an important role in biogeochemical cycles and the mineralization of pollutants. Fungi, bacteria, archaea and yeasts have been isolated in every Antarctic habitat, including lakes, ponds, rivers, streams, rocks, and soils [2, 3].

Several studies dealing with bacterial diversity and bioprospecting for cold-active hydrolytic enzymes from culturable bacteria of various Antarctic ecosystems have been performed. These have shown that these extreme environments provide a good setting for screening this sort of enzyme with potential biotechnological applications and economic benefits [4–7].

The agro and food industry generates lots of solid wastes (chitinous, lignocellulosic, and keratinous biomass) which may suitably feed into the economy with a novel valorization approach. Keratin is an insoluble fibrous protein with a recalcitrant structure. After cellulose and chitin, it is the third most abundant polymer in nature. It is the structural component of hair, feathers, hooves, nails, horns, beaks, fish teeth, and the reptilian osteoderm of a wide spectrum of vertebrates [8]. It is resistant to proteolysis by common proteases due to its tightly packed, supercoiled polypeptide chains that are characterized by hydrogen bonds, hydrophobic interactions, and a high level of crosslinking [8]. The slaughterhouses, leather industries, and poultry processing farms are some of the sectors that generate keratinous biomass in large quantities. The accumulation of the agro-industrial wastes results in varying degrees of pollution in the environs [9].

Keratinases (E.C. 3.4.99.11) are a class of proteolytic enzymes produced by certain fungi, bacteria, and yeasts. These enzymes can hydrolyze keratinous substrates such as wool, nails, feathers, and hair waste from tanneries. They are characterized by their preference to aromatic and hydrophobic residues at the P1 position. Based on their catalytic type, they can be classified as metallo, serine or metallo-serine proteases [10, 11].

Most keratinases described to this day are produced by mesophilic and thermophilic organisms [12–14]. To our knowledge, there are only a few reports dealing with Antarctic psychrotolerant feather-degrading bacteria [15, 16]. Just as cold microbial proteases, commercial value of cold-active keratinases is of great potential, mainly due to their high stability and activity in harsh operational conditions [16, 17].

The microbial valorization of keratinous waste biomass may yield high-quality protein hydrolysates [18], organic fertilizer [19], peptone substituted microbial growth media [20], plant growth hormones [21], and industrially important enzymes [12].

Consequently, the exploration and exploitation of microbial diversity for novel keratinolytic potentials remains topical. All the identified microbes associated with keratolysis are, either bacteria or fungi [22] Being bacteria the most viable microbe for keratinous waste bioconversion. Autochthonous and/or allochthonous bacterial strains from different ecological niches have shown to possess potential for the bioconversion of keratinous biomass into value-added products.

A continuum in the discovery of keratinases with novel properties and functions would, significantly, revolutionize the bio-economy landscape, and the prospects presented by proteolytic enzymes [23].

In the present study, we report the isolation and characterization of the psychrotolerant keratinolytic bacterial strain *Pedobacter* sp. 3.14.7 (MF 347939.1). As far as we know, this is the first report of the genus *Pedobacter* having the ability to degrade chicken feathers and express a special class of proteases: keratinase. Biochemical characterization of the keratinolytic extract and its potential as a detergent additive is presented.

## Methods

### Isolation of microorganisms

Culturable bacteria from a feather-degrading consortium were isolated from feather samples of *Chionis alba* (*paloma antárctica* or snowy sheatbill) collected from nests near Collins Glacier (coordinates UTM62.184579 S, 58.869612 O) in March 2016. Bits of feathers were collected aseptically and preserved at 4 °C until processing.

In order to obtain the consortium, 1 g of the sample was suspended in a 100 ml Erlenmeyer flask containing 20 ml of sterilized distilled water and incubated at 150 rpm and 15 °C for 30 min. After that, 1 ml of the supernatant was transferred into a 250-ml Erlenmeyer flask containing 50 ml of feather mineral liquid medium (FMM) composed of (g.l^−1^): K_2_HPO_4_ 2.486, NaH_2_PO_4_ 0.496, MgCl_2_ 0.01, FeCl_3_ 0.016, CaCl_2_ 0.0001, ZnCl_2_ 0.013, and 10 g of chicken feathers (washed and defatted) per liter of mineral liquid medium; this solution had a pH of 6.0. Cultures were carried out at 15 °C under 180 rpm agitation until complete feather degradation was observed. After four sequential transfers into FMM, a keratinolytic consortium was obtained and preserved in a 15% v v^−1^ glycerol solution at −80 °C.

In order to isolate culturable bacteria, 100 μl of the final consortium and serial dilutions (up to 10^−9^) were streaked on Nutrient Agar plates (NA). After one week of incubation at 15 °C, visible morphological types of single colonies were isolated, transferred into new Nutrient Agar plates and kept at 4 °C and transferred monthly. Additionally, isolates were kept frozen in a nutrient broth supplemented with a 10% v v^−1^ glycerol solution at −80 °C for long-term preservation.

Isolated strains were stored in the Microbiological Culture Collection of CINDEFI-CONICET Institute and in the Cátedra de Microbiología, Facultad de Química, UdeLar Culture Collection (Montevideo, Uruguay).

### Screening of proteolytic and keratinolytic activity

For primary screening of proteolytic activity, isolates were inoculated in skimmed milk agar plates (5 g−l^−1^ skimmed milk and 15 g l^−1^ agar) and incubated at 15 °C for one week. Zones of clearing around the colonies were used as criteria for selecting proteolytic strains.

Then, proteolytic bacteria were cultured in 250-ml Erlenmeyer flasks containing 50 ml of FMM. Cultures were conducted for one week at 15 °C under 180 rpm agitation. The most effective feather-degrading isolate—the first one that fully degraded chicken feathers—was selected for further studies.

### Molecular identification

The genomic DNA of the selected isolate was extracted using the boiling method [24], and it was identified using the 16S rDNA genes sequence analysis. This product was amplified applying the PCR method with the following universal primers, 27 F primer (5′-AGAGTTTGATCCTGGCTCAG-3) and 1387 R primer (5´- GGGCGGWGTGTACAAGGC-3´) [25], following the standard procedure. PCR conditions were as follows: initial denaturation at 95 °C for 5 min, followed by 38 cycles of denaturation at 95 °C for 1 min, an annealing temperature of 46 °C maintained for 1 min, and final extension at 72 °C for 1 min [25]

The amplicon was purified using a PCR purification kit (Qiagen, Germany) and it was sequenced by Macrogen, a sequencing service from South Korea using the Sanger-sequencing method. The nucleotide sequence was compared with type sequences from the National Center for Biotechnology Information (NCBI) GenBank database using the BLAST program. A phylogenetic tree was developed using the Molecular Evolutionary Genetics Analysis package (MEGA version 6.0 [26],). The sequences were aligned using the ClustalW function and the Maximum Likelihood phylogenetic tree was created employing the Kimura 2-parameters algorithm [26, 27]. The robustness of the phylogeny was tested with a bootstrap analysis of 1000 iterations [28]. The nucleotide sequence was submitted to the GenBank database of the National Centre for Biotechnology Information (NCBI) under accession number MF 347939.1.

### Growth kinetics at different temperatures

The effect of temperature on growth kinetics of *Pedobacter* sp. 3.14.7 was analyzed in 250-ml Erlenmeyer flasks containing 50 ml of Nutrient Broth medium. Nutrient Broth mediums were inoculated with an overnight seed culture (OD_i600_=0.05). These cultures were incubated in rotary shakers for 48 h at 180 rpm, and 15 °C, 20 °C, and 28 °C. Samples were withdrawn aseptically at different intervals and growth was estimated spectrophotometrically at 600 nm. OD was determined in a T60 UV-Visible spectrophotometer (PG Instruments, UK). Maximum growth rates (μ_max_) at different temperatures were estimated from microbial growth curves applying the accumulation balance for *batch* cultures and the Monod equation. The degradation of feathers was also assessed at these temperatures.

### Culture conditions for keratinases production and enzymatic assays

Production of keratinases from *Pedobacter* sp. 3.14.7 was performed in 250-ml Erlenmeyer flasks containing 50 ml of FMM containing 30 g l^−1^ of chicken feathers. Cultures were performed in a rotary shaker for seven days at 20 °C and 180 rpm, and samples were withdrawn periodically. Culture supernatants were obtained after centrifugation at 10,000*g* for 10 min.

Proteolytic activity was assessed using azocasein (Sigma Aldrich, USA) as substrate. In brief, 100 μl of suitable diluted enzyme was added to 250 μl of azocasein solution (10 g l^−1^ Tris-HCl buffer 20 mmol.l^−1^; pH 7.5). After incubation at 37 °C for 60 min, reaction was stopped by adding 1000 μl of trichloroacetic acid (TCA; 10%, w v^−1^). The admixture was centrifuged (10,000 *g*, 5 min), and the resulting supernatant (500 μl) was added to 500 μl of NaOH (1.0 mol l^−1^). Absorbance (Abs) was measured at 440 nm. Assays were performed in triplicates and suitable controls were prepared by adding TCA to the reaction mixtures before adding the enzyme. One unit (U) of protease activity was arbitrarily defined as an increase of 0.01 absorbance unit under the experimental conditions used.

Keratinolytic activity was assayed using an azokeratin substrate following the protocol described by Moridshahi [29]. In brief, a reaction admixture containing 100 μl of suitable diluted enzyme and 30 mg of azokeratin in 800 μl of 20 mM Tris-HCl buffer at pH 7.5 was incubated for 60 min at 37 °C. This reaction was then stopped by adding 200 μl of 10% (w v^−1^) TCA, and the admixture was centrifuged at 10,000*g* for 10 min. Absorbance of the supernatant was measured at 440 nm. One unit of keratinolytic activity was arbitrarily defined as an increase of 0.01 absorbance unit under assay conditions.

### Feather substrate loss

Feather degradation was studied by using the weight loss method [30]. Substrate loss was determined after separation of the residual substrate from the fermented broth using Whatman filter paper Grade 1. The collected feathers that did not degrade were rinsed several times with distilled water and dried at 105 °C. The dry weight of un-degraded feathers was measured and expressed as a percentage of feather degradation by this formula:

$$\mathrm{Percentage}\ \mathrm{of}\ \mathrm{feather}\ \mathrm{degradation}\ \left(\%\right)=\mathrm{F}/\mathrm{W}\times 100$$

where *F* is the weight of un-degraded feathers after fermentation and *W* is the total feather weight before fermentation. The experience was performed in triplicate and results were expressed as substrate loss ± SD.

### Protein determination

Protein concentration was determined using the method described by Bradford [31] using bovine serum albumin (Sigma-Aldrich) as a standard.

### Biochemical properties

#### Effect of pH on activity and stability

Optimum pH of keratinase was studied over a pH range of 6.0–12.0 at 37 °C using azocasein as substrate, while its stability was measured incubating the enzyme at different pHs (MES-Tris-Glycine buffers, 20 mM each, ranging from 3.0 to 12.0) for 1 h at 37 °C. After the incubation period, residual enzyme activity was analyzed under standard assay conditions using azocasein as a substrate.

#### Effect of temperature on activity and stability

Optimum temperature for keratinase activity was assessed over the range of 20–75 °C and its stability was examined at different temperatures (20–65 °C) by incubating it for different time intervals (15–60 min). After the incubation period, residual activity was measured under enzyme assay conditions. The activity of enzyme that was not subjected to heat was considered as an activity of 100%.

#### Effects of protease inhibitors and metal ions on keratinase activity

The protease inhibitors phenylmethylsulphonyl fluoride (PMSF, 1 mM), ethylenediaminetetraacetic acid (EDTA, 1 mM), 1,10-phenanthroline (1 mM, iodoacetamide (1 mM), and pepstatin A (5 μM) were tested on enzyme activity in order to determine which protease type is present in the crude extract. A volume of 100 μl of crude extract was incubated for 1 h in the presence of each inhibitor, and proteolytic activity was then determined as in the procedure described above. Enzyme activity in the absence of these inhibitors was considered as an activity of 100%.

The different mono and divalent metal ions listed in Table 1 were incubated with enzyme crude extracts at room temperature for 1 h in order to study their effect on enzyme activity. After an incubation period of 1 h, residual activity was assessed. The activity in the absence of metal ions was considered as an activity of 100%.

**Table 1:** Biochemical properties of *Pedobacter* sp. 3.14.7 keratinase in comparison with other keratinases reported in bibliography

Microorganism	Optimum pH	Optimum temperature (°C)	pH range	pH stability	Temperature stability (°C)	Inhibitors	Reference
*Pedobacter* sp. 3.14.7	7.5	55	6.0–8.0	7.0–10.0	30% residual activity (1 h at 55 °C)	EDTA phenanthroline	This work
*Pedobacter cryoconitis*	8.0	40	7.0–10.0	7.0–9.0	T1/2 30 min (40 °C)	EDTA phenanthroline	[32]
*Bacillus zhangzhouensis*	9.5	60	7.0–10.5	7.0–10.5	T1/2 120 min (60 °C)	Co^+2^, Fe^+2^, Hg^+2^ PMSF	[29]
*Bacillus pumilus*	9.0	45	7.0–10.0	NR	NR	Zn^+2^, Hg^+2^, Pb^+2^ PMSF	[11]
*Ochrobactrum intermedium*	9.0	40	4.0–9.0	9.0 (5h)	40–60 almost 2 h	Zn^+2^, Fe^+2^, Cu^+2^, Mn^+2^, Ag^+2^, Hg^+2^ PMSF, EDTA	[30]
*Caldicoprobacter algeriensis*	7.0	50	2.0–10.0	5.0–9.5	T1/2 120 h (50 °C)	Ni^+2^, Hg^+2^, Cd^+2^ PMSF	[33]

*NR* not reported, *t1/2* half-life of the enzyme, *EDTA* ethylenediaminetetraacetic acid, *PMSF* phenylmethylsulphonyl fluoride

#### SDS-PAGE analysis and zymography

Casein zymography was performed along with sodium dodecyl sulfate polyacrylamide gel electrophoresis (SDS-PAGE) following the protocol described by Delgado-García et al. [34] with slight modifications. In brief, after electrophoresis, the gel was treated with a solution of Triton X-100 (2.5% wv^−1^) for 1 h. This gel was rinsed twice with distilled water in order to remove the Triton X-100 present. It was then incubated with Tris-HCl buffer (20 mM, pH 7.5) containing 1% wv^−1^ casein for 1 h. The gel was stained with Coomassie Brilliant Blue G-250 Coomassie Colloidal method [35]. Development of zones of clearing on the gel’s blue background indicated the presence of protease activity.

Pierce TM Unstained Protein MW (# 26610) was used as a molecular mass marker.

### Potential of *Pedobacter* keratinase as detergent additive

#### Stability of protease from *Pedobacter* sp. 3.14.7 in the presence of bleaches and surfactants

Using the procedure previously described, the enzyme was pre-incubated for 1 h at room temperature in the presence of several surfactants (Triton X-100, Tween 20, and SDS) and oxidizing agents (hydrogen peroxide and sodium perborate). Residual activity was measured as it was stated before. The activity of the enzyme pre-incubated without any additives was considered as an activity of 100%.

#### Stability and compatibility of protease in the presence of laundry detergents

Stability and compatibility of the keratinase produced by *Pedobacter* sp. 3.14.7 with solid and liquid laundry detergents were assessed by pre-incubating the enzyme preparation for 1 h at 30 °C and 40 °C. Residual activities were determined under standard assay conditions. The enzyme activity of a control sample (without any detergent), incubated under the same conditions was considered as 100%. Detergents were heat-inactivated (1 h at 65 °C) before adding the enzyme in order to inactivate endogenous enzymes.

Solid detergents were diluted in tap water to give a final concentration of 7 mg ml^−1^, and liquid detergents were diluted 100-fold to simulate washing conditions.

#### Washing performance

Blood-stained-clean cotton cloth pieces (2.5 cm × 2.5 cm) were subjected to wash simulation treatment in order to determine the efficiency of *Pedobacter’*s keratinase as biodetergent additive. The endogenous proteases contained in Skip liquid detergent were inactivated by heating the diluted detergent for 1 h at 65 °C prior the addition of *Pedobacter* crude extract.

Two stained cloth pieces were taken in separate flasks, with 50 ml as the final volume, as indicated above: flask with tape water, only, flask with tap water and commercial detergent at the final concentration of 7 mg ml^−1^, and flask with tap water, commercial detergent, and crude enzyme of *Pedobacter* (10.5 UC/50ml). Each flask was incubated at two temperatures: 20 and 50 °C for 40 and 80 min under agitation (200 rpm). After incubation, cloth pieces were taken out, rinsed with water, and dried. Visual examination of various pieces showed the effect of the crude enzyme in the removal of stains.

### Statistical analysis

Results were expressed as residual activity (%) ± SD, and they were obtained by averaging three independent experiments.

## Results

### Isolation and screening for feather-degrading and keratinase-producing bacteria

In the present study, seven culturable bacteria from a feather-degrading consortium were isolated. All of them developed proteolytic activity in milk agar and visibly degraded feathers in FMM. Among these keratinolytic bacteria, isolate 3.14.7 was selected due to the degradation rate it displayed at 20 °C. After 7 days, a complete degradation of the feather sample was observed.

### Identification of selected bacterium

**Fig. 1:**
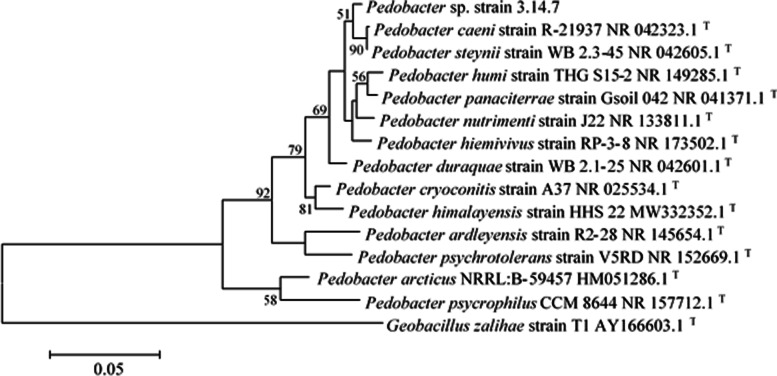
Phylogenetic tree of *Pedobacter* sp. 3.14.7 (MF 347939.1) within the relative strains of genus *Pedobacter*. The tree was created using the Maximum Likelihood tree method (K2P distance method). Bootstrap values (1000 tree interactions, shown as %) ≥ 50 are indicated at the nodes. *Geobacillus zalihae* strain T1 was considered as an out of group bacterium

Based on the nucleotide sequence of a preserved segment of the 16S rRNA gene, the bacterium was identified as *Pedobacter* sp. and named *Pedobacter* sp. 3.14.7. The strain 3.14.7 showed 99.22% homology with *Pedobacter steynii* WB2.3–45 and 99% with *P. caeni* R-21937. Based on its 16S rRNA sequence and its position in the phylogenetic tree (Fig. 1), this bacterium was taxonomically closer to *P. steynii* and *P. caeni* than to *P. cryoconitis*, *P. psychrophilus*, and *P. ardleyensis*.

**Fig. 2:**
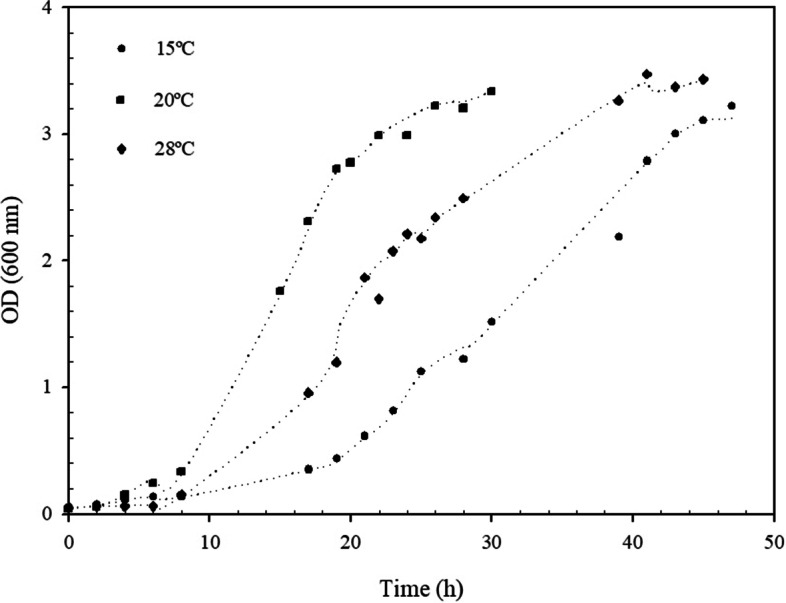
Effect of temperature on *Pedobacter* sp. 3.14.7 growth on Nutrient Broth medium; cell growth was monitored by measuring its optical density at 600 nm

This strain was able to grow at temperatures ranging from 4 to 28 °C, with the maximum growth rate occurring at 20 °C (μ_max_=0.23 h^−1^). At lower temperatures (4-15 °C), growth was relatively slow (μ _max_=0.11 h^−1^ at 15 °C), and the cell density reached its maximum at 47 h; *Pedobacter* sp. 3.14.7 displayed the same behavior when it was grown at 28 °C. At this temperature, its μ _max_ was 0.19 h^−1^, and the maximum cell density was reached at 42 h (Fig. 2). According to the most widely accepted definition [36], it can be assumed that the isolate displayed a psychrotolerant growth pattern.

**Fig. 3:**
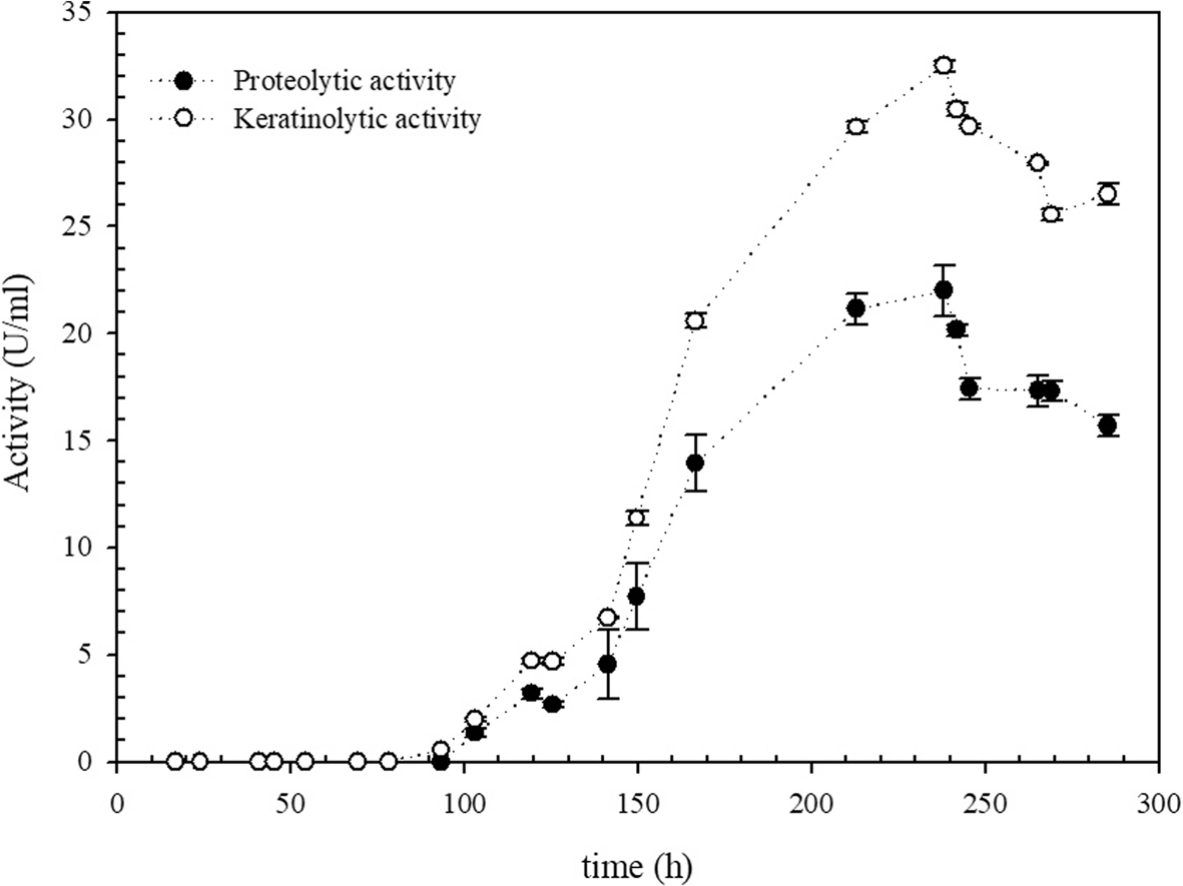
Time course of production of protease and keratinase expressed by *Pedobacter* sp. 3.14.7

It has been reported that keratinolytic and proteolytic activity are linearly related and thus, proteolytic activity can be used indirectly to measure keratinolytic activity [37]. At 15 °C, a small degree of enzymatic activity was detected. At 20 °C, complete feather degradation was observed, along with the maximum enzymatic activity. After a cultivation period of 238 h, maximum proteolytic (22.00 ± 1.17 U ml^−1^) and keratinolytic (33.04 ± 1.09 U ml^−1^) activities were achieved (Fig. 3). At this temperature, a feather sample conversion of approximately 85 ± 1.5% was achieved.

At higher cultivation temperatures (28 °C), even though the strain grew well in nutrient broth, no degradation of feathers was observed in FMM supplemented with feathers.

### Biochemical characterization

*Pedobacter* sp. 3.14.7 was able to degrade raw feathers and produce a proteolytic/keratinolytic crude extract.

**Fig. 4:**
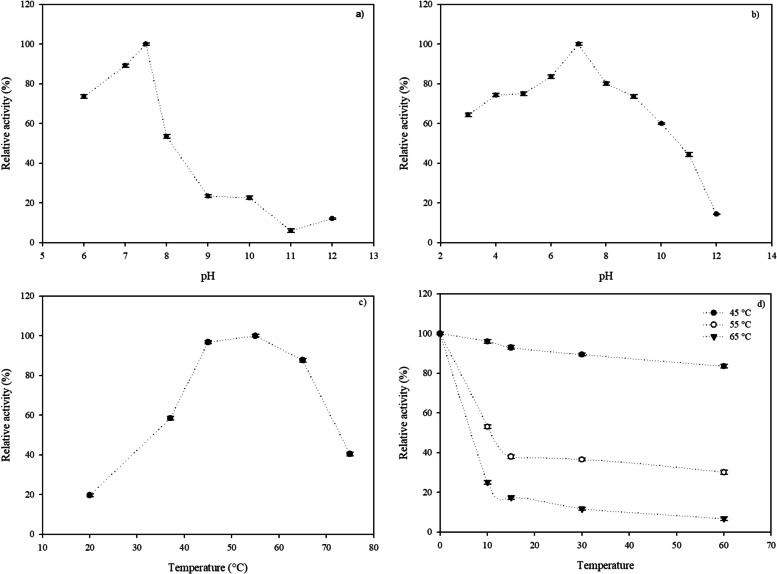
Effect of temperature and pH on the activity of keratinase expressed by *Pedobacter* sp. 3.14.7; **a** effect of pH on keratinase activity; **b** pH stability of keratinase enzyme. The enzyme was pre-incubated at different pH values for 1 h at 37 °C. Then, the residual activity was measured under standard assay conditions; **c** effect of temperature on keratinase activity; **d** thermal stability of keratinase enzyme. The enzyme was pre-incubated at different temperatures and then the residual activity was determined under standard assay conditions.; values represent the means ± SD of three replicates

Crude extract was active over a broad pH range, displaying a higher activity in the pH range of 6.0–8.0; being its optimal pH 7.5 (Fig. 4a). About 70% of the maximum activity was measured at pH 6.0. Only 22% of the maximum activity was measured at pH 10.0.

Concerning to pH stability, crude extract was 100% stable at pH 7.0 when it was incubated for 1 h at 20 °C, while it presented 80% of residual activity at pH 8.0–9.0, and 60% at pH 10.0 (Fig. 4b).

The optimum temperature for protease activity at pH 7.5 was 55 °C (Fig. 4c). An increase in temperature significantly reduced enzymatic activity; total inactivation occurred at 85 °C. In the 20–45 °C temperature range, the residual activity measured ranged from 15 to 98%.

Using the Arrhenius plot’s linear part, the apparent activation energy for the hydrolysis of azocasein by the cold-active protease from *Pedobacter* sp. 3.14.7 was calculated as 35.3 kJ mol^−1^.

Thermostability studies have shown that crude extract was 100% stable up to 37 °C for 1 h, and a decrease of thermal stability was detected when the incubation temperature was raised beyond that threshold. Crude extract retained an activity of about 83% after an incubation period of 1 h at 45 °C, indicating that its stability is still good at high-moderate temperatures. The enzyme was rapidly inactivated at temperatures beyond 45 °C. After an incubation period of 1 h at 55 °C, only 30% of enzyme activity remained, while incubation at 65 °C resulted in a loss of 93% of initial activity (Fig. 4d).

The relative inhibitory effects of well-known inhibitors are listed in Table 2. As it can be seen, enzyme activity was strongly inhibited by the metal chelating agents EDTA and phenanthroline (general inhibitors of metalloproteases), suggesting that this enzyme belongs to the metalloprotease group.

**Table 2:** Effect of chemical agents on keratinase activity. Values are the mean of three replicates ± standard deviation. 100% of activity corresponds to those displayed by the enzyme without the presence of any chemical

	Concentration	Residual activity (%)
Chemical		
None		100
Inhibitor		
PMSF	1 mM	103.1 ± 5.8
Iodoacetate	10 mM	95.5 ± 6.3
EDTA	5 mM	33.5 ± 1.8
1,10-Phenanthroline	1 mM	83.3 ± 3.4
Pepstatin A	100 μg/ml	108.1 ± 7.3
Metal ion		
Mg^2+^	1 mM	111.8 ± 6.0
Zn^2+^	1 mM	107.6 ± 1.8
Ca^2+^	1 mM	104.6 ± 1.2
Hg^2+^	1 mM	95.9 ± 3.6
Co^+2^	1 mM	97.3 ± 0.6
Mn^+2^	1 mM	112.8 ± 4.3
Na^+^	1 mM	90.3 ± 0.6
K^+^	1 mM	89.9 ± 1.2
Detergents		
Triton X-100	0.5 % (v/v)	97.7 ± 2.7
Tween 20	0.5 % (v/v)	98.5 ± 2.0
SDS	0.5 % (v/v)	75.9 ± 3.4
Bleaching agent		
H_2_O_2_	1 % (w/v)	100.9 ± 2.6
	2 % (w/v)	98.9 ± 2.5
	3 % (w/v)	79.8 ± 5.5
Sodium perborate	0.2 % (w/v)	86.6 ± 5.1
	0.5 % (w/v)	80.3 ± 5.1
Solvent		
DMSO	1 % (v/v)	100 ± 0.6
Ethanol	1 % (v/v)	100 ± 0.3
Methanol	1 % (v/v)	100 ± 0.6
Isopropanol	1 % (v/v)	100 ± 1.0

Concerning the effect of various metal ions on enzyme activity (Table 2), it was seen that the presence of Mg^+2^, Zn^+2^, Ca^+2^, and Mn^+2^ slightly enhanced it, while the remaining metal ions studied slightly inhibited it. None of the metal ions tested caused total inhibition; not even Hg_2_^+2^, which is one of the metals known to affect keratinolytic enzymes this way.

Presence of Triton X-100 and Tween 20 slightly inhibited activity, while presence of SDS resulted in an inhibition of 24.1% in enzymatic activity. Biochemical properties of *Pedobacter* sp. 3.14.7 keratinase in comparison with other keratinases reported in bibliography are presented in Table 1.

**Fig. 5:**
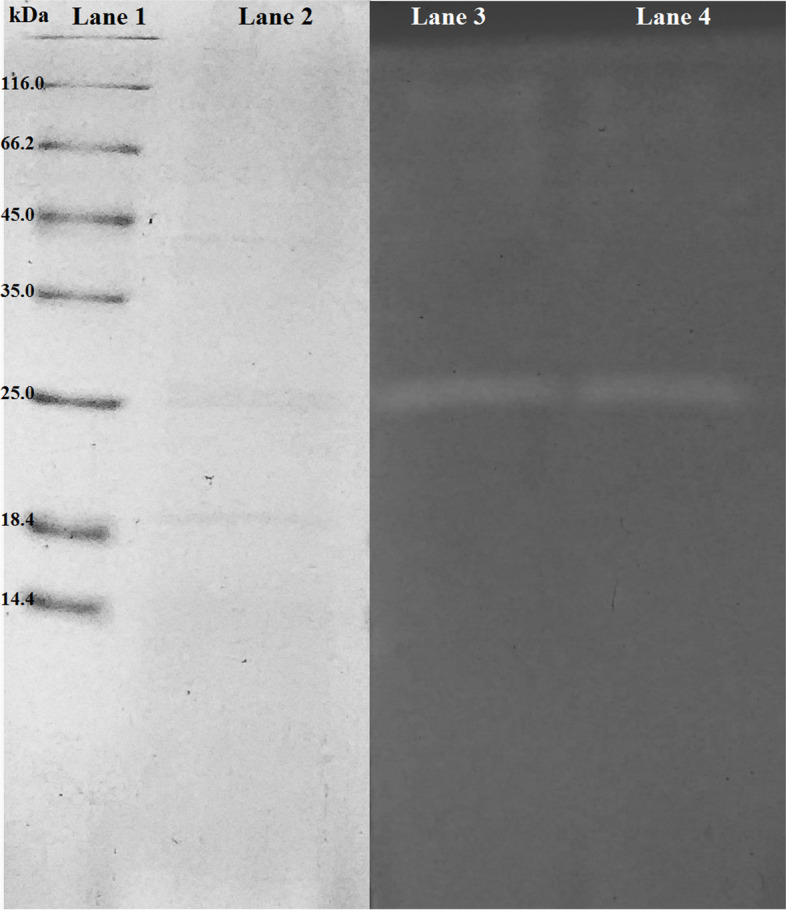
SDS-PAGE and zymogram analysis of keratinase produced by *Pedobacter* sp. 3.14.7; lane 1: standard pattern of protein markers (kDa, Thermo Scientific™ Pierce™ Unstained Protein 26610); lane 2: protein pattern of crude keratinase; lanes 3 and 4: zymogram staining of the keratinase

SDS-PAGE analysis showed that the crude extract displayed one band corresponding to an apparent molecular mass of 25 kDa; an activity gel analysis confirmed that the protease with keratinolytic activity produced by *Pedobacter* sp. 3.14.7 is a single monomeric protein (Fig. 5).

### Potential of *Pedobacter* keratinase as detergent additive

Table 2 shows that keratinase produced by *Pedobacter* sp. 3.14.7 displayed little enzyme activity inhibition in the presence of oxidizing agents: in the presence of 3.0% of H_2_O_2_ its activity loss was only 20.2% (it retained 79.8 ± 5.5% of its initial activity after incubation for 1 h at 20 °C), and in the presence of 0.5% sodium perborate, it lost 19.7% (it retained 80.3 ± 5.1%). These are very important features for potentially using the enzyme in detergent formulation, in addition to its stability and compatibility with different laundry detergents.

Data listed in Table 3 show that crude extract displayed a high stability in the presence of all solid laundry detergents tested when incubated at 30 and 40 °C, retaining between 60% (Drive detergent at 40 °C) and 86% (Skip detergent at 40 °C) of its activity after 1 h of incubation.

**Table 3:** Stability and compatibility of keratinase in presence of different commercial detergents. Values are the mean of three replicates ± standard deviation. 100% of activity corresponds to those displayed by the enzyme without the presence of any detergent

Chemical	Residual activity (%)	
	30°C	40°C
*Solid detergents*		
Drive	102.5 ± 1.22	61.4 ± 1.4
Ariel	108.3 ± 2.08	100.0 ± 1.2
Ace	100.0 ± 1.71	86.4 ± 1.78
Skip	98.6 ± 1.84	93.15 ± .61
*Liquid detergents*		
Ace	112.6 ± 3.5	107.8 ± 2.1
Ala	112.2 ± 2.9	104.3 ± 1.8
Ariel	105.5 ± 2.3	88.5 ± 1.4
Skip	104.8 ± 3.6	98.2 ± 1.5

Even better stability was observed with liquid detergents, with a residual activity of 100 ± 0.14% at 30 °C, and 88.5 ± 0.16% at 40 °C, respectively, using Ariel detergent (Table 3).

**Fig. 6:**
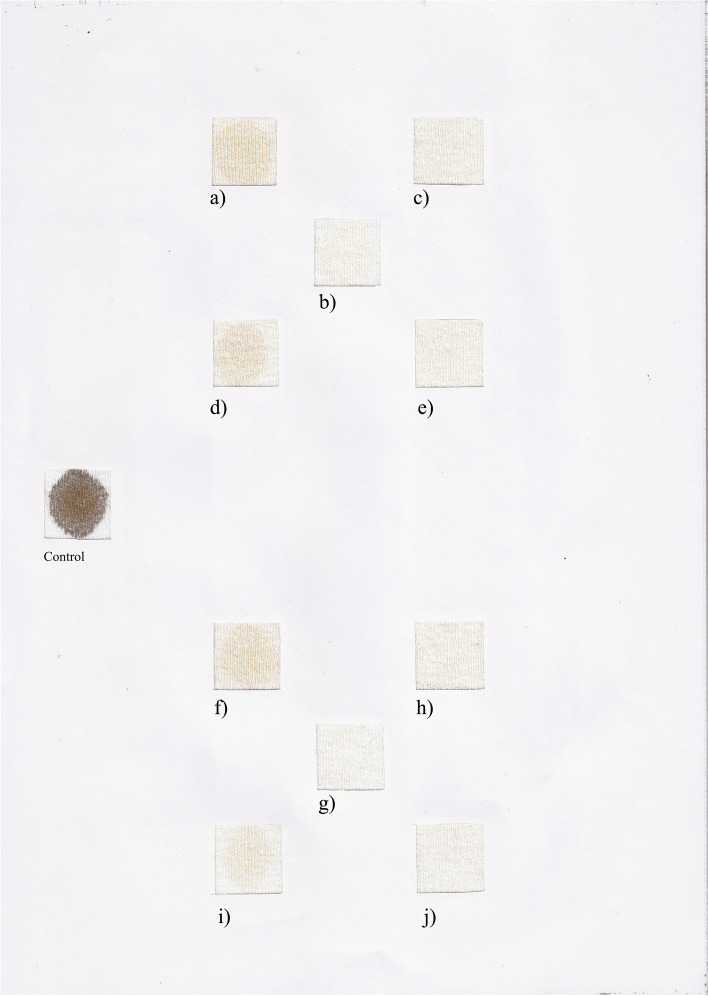
Washing performance analysis of the *Pedobacter* sp. 3.14.7 enzyme preparation in the presence of the commercial detergent Skip. Analysis was done at 20 °C (**a**–**e**) and 50 °C (**f**–**j**), for 40 min (**a**–**e** and **g**–**i**) and 80 min (**f**–**j**). Cloth washed with tap water (**a**, **d**, **f**, and **i**); **b**, **g** cloth washed with Skip. In **c**, **e**, **h**, and **j**, the cloth was washed with endogenous-enzyme inactivated Skip, supplemented with the crude enzyme with keratinase enzyme

Figure 6 shows the wash performance of *Pedobacter* sp. 3.7.14 proteolytic/keratinolytic crude extract at two different temperatures and times (20 and 50 °C for 40 and 80 min). It could be seen that similar results were obtained at 20 and 50 °C when the commercial detergent skip was supplemented with our crude extract and blood stains were completely removed. Increasing time wash and temperature did not enhance blood removal.

## Discussion

Although Antarctica has been classified as one of the harshest environments in the world, a lot of reports have demonstrated that this continent is plentiful of microorganisms. Microorganisms have been isolated in every ice-free Antarctic habitat, been bacteria the dominant group [38–41].

The bioprospection of cold active hydrolytic enzymes produced by Antarctic bacteria is a topic that increases every year and demonstrates the need to characterize not only the producer microorganisms but also the enzymes that they produce in order to find new biocatalysts. The aim of our work is to go deeper in the study of Antarctic proteases with keratinolytic activity. This type of enzyme is attractive because it can be applied in various industries, such as leather, textile, chemical, pharmaceutical, food, and nutrition industries, as well as in biotechnology protection [42].

The *Pedobacter* genus in the Sphingobacteriaceae family is identified as Gram-negative, aerobic and rod-shaped bacteria [43]. To our knowledge, seven *Pedobacter* species from Antarctica have been isolated: *P. ardleyensis* R2-28 [44], *P. psychrophilus* CCM8644 [45], *P. cryoconitis* PAMC 27485 [46], *P. jamserossensis* CCM8689, *P. lithocola* CCM 8691, *P. mendelii* CCM 8685 and *P. petrophilus* CCM 8687 [47]. The level of sequence similarity with these strains ranges from 93 to 97%. On R2A, all these species except for *P. cryoconitis* have a reddish-pink-deep red color, while *P. cryoconitis* and *Pedobacter* sp. 3.14.7 develop creamy white colonies. Our group reported for the first time one species of *Pedobacter* as keratinolytic. In a previous work, we reported the optimization of the production of its proteases with keratinolytic activity and the production and characterization of the feather hydrolysates as consequence of this keratinolytic activity [48]. Here, we report the characterization of this proteolytic extract and its potential use as a bioactive component of detergents.

Although *Pedobacter* sp. 3.14.7 grows at 28 °C, it demonstrates a psychrotolerant growth pattern with no keratinolytic activity at this temperature (28 °C). This behavior could be related with the thermal inhibition of the enzymes. Thermal inhibition of extracellular enzyme production is a common feature of cold-adapted microorganisms [32]. Chen et al. [49] proposed that considering the permanently cold natural habitat of these strains, a decrease in proteolytic activity could be explained by the fact that some internal regulatory factors which control this enzymatic expression or transport may be unstable at temperatures over 25 °C.

El-Refai et al. [50] reported different degrees of feather degradation by different *Bacillus* spp. at 37 °C. The extent of feather degradation varied between 42.0% for *B. subitilis* to 96% for *Bacillus pumilus* FH9. In the case of *B. licheniformis* SA1, feather degradation reached a value of 70.8%. After 6 days of incubation at 37 °C, *Bacillus cereus* KB043 achieved a weight loss of 78.16% [51]. As can be seen, *Pedobacter* sp. 3.14.7 is a relatively more efficient feather-degrading strain, and it has the advantage that degradation is achieved at room temperature (20 °C). A similar result (degradation of 86%) was achieved with *Pseudomonas* sp. LM19 in optimized conditions as it was reported by Mohamad et al. [52].

While most cold-active proteases display optimum activity at 30-45 °C, there are some reports of psychrophilic bacteria producing proteases with optimum activity in the 50-60 °C temperature range [53, 54]. The cold-active metalloprotease from *Pedobacter cryoconitis* reported by Margesin et al. [32] differs from the one reported in this study (optimum pH 7.5 and optimum temperature 55 °C) by displaying an optimum activity at pH 8.0 and 40 °C. Pereira et al. [17] reported the isolation of three novel Antarctic feather-degrading bacteria: *Lysobacter* sp. A03, *Arthrobacter* sp. A08 and *Chryseobacterium* sp. A17U. These keratinases have too an optimal temperature range of proteolytic activity of about 35–40 °C; their optimal activity occurs in alkaline conditions (pH 8.5-9.5). At pH 5.5–6.5, the three isolates displayed less proteolytic activities.

Adaptation of organisms to cold temperatures is reflected by a low activation energy required for enzymatic substrate hydrolysis [32, 55]. Using the Arrhenius plot’s linear portion [56], the apparent activation energy for the hydrolysis of azocasein by the cold-active protease from *Pedobacter* sp. 3.14.7 was calculated as 35.3 kJ mol^−1^, whereas Subtilisin C and Savinase displayed activation energies of 59.1 and 62.2 kJ mol^−1^, respectively. Only a small number of studies have determined the activation energy (Ea) of enzymes found in soils, and even fewer studies have measured the Ea in Arctic and Tropical soils, or in subsurface soils. Steinweg et al. [57] determined the Ea of four typical lignocellulose-degrading enzymes from various soils, including Arctic and Subarctic soils: the Ea found for b-glucosidase is similar to the Ea estimated in this study (35.4 and 36.5 kJ/mol for Arctic and Subarctic soils, respectively.)

Thermostability studies demonstrate that crude extract produced by *Pedobacter* sp. 3.14.7 is more stable than the metalloprotease reported by Margesin et al. [32], which retained an activity of 50% after 30 min of incubation at 40 °C; it was completely inactivated at 50 °C.

As keratinases are a special kind of proteases one of the rutinary study is to see its sensitivity to proteases inhibitors. The inhibition behavior in front of the metal-chelating agents EDTA and phenanthroline suggest that this enzyme belongs to the metalloprotease group. Although a few bacterial metalloproteases with keratinolytic activity have been reported, the majority of known keratinases are endopeptidases belonging to the serine protease family [58]. Among Antarctic keratinolytic bacteria, *Lysobacter* sp. A03 was reported to produce serine proteases, while the strains *Arthrobacter* sp. A08 and *Chryseobacterium* sp. A17U had their proteolytic activity inhibited by EDTA, and partially inhibited by 1,10-phenanthroline, indicating thus their metalloprotease nature [17].

SDS is a strong anionic surfactant that has been reported to be inhibitory of various proteases and keratinases [59, 60]. The SDS inhibition on *Pedobacter* sp. 3.14.7 was only of 24.1% (75.9 % of residual activity) being lesser than the inhibitory effect that this compound exerts on the protease expressed by *Pedobacter* cryoconitis A37T, only 15% of proteolytic activity remained after 1 h of incubation at 20 °C in the presence of 0.1% of SDS [32].

The apparent molecular mass of 25 kDa that presents the protease with keratinolytic activity of *Pedobacter* sp. 3.14.7 is in accordance with that of several reported keratinases such as those from *Brevibacillus parabrevis*, which produces a metallo-serine keratinase with a molecular mass of about 28 kDa [61], and *Acinetobacter sp*. R-1 [62]. Metalloproteases with similar molecular mass are also produced by the cold-adapted bacteria *Pseudomonas strain* DY-A [63] and *Pedobacter cryoconitis* [32]. Higher molecular masses have been reported for keratinases with metalloprotease characteristics: the thermotolerant, alkalotolerant metallo-keratinase from *Bacillus subtilis* NRC3 is a monomeric protein with a MW of 32 kDa [58]; *Streptomyces aureofaciens* K13 produces a 46-kDa metallo-serine keratinase [64].

One of the common ingredients of modern bleach-based detergents is hydrogen peroxide, which makes many of the available proteases undergo oxidative inactivation. A relative resistance to SDS and bleaches is one of the properties sought for in new proteases and their industrial applications in detergent formulation. In the presence of 3.0% of H_2_O_2_ and, in the presence of 0.5% sodium perborate little enzyme activity inhibition was observed. These characteristics are very important for its potentially use in detergent formulation, in addition to its stability and compatibility with different laundry detergents*. Pedobacter’*s crude extract displayed a high stability in the presence of all solid and liquid laundry detergents tested, proving to be more stable than the alkaline protease reported by Singh et al. [65] which retained 37% of its initial activity after 1 h of incubation at 40 °C in the presence of 5 mg/ml of Ariel detergent.

Stability studies of *Bacillus pumilus* GRK, Ramakrishna Reddy et al. [66] have reported a residual activity of 89.48 ± 0.22% of using Ariel detergent. These results are in accordance with those presented by several authors [67–70] and support the use of this kind of enzyme in industrial applications as a cleaning bioadditive in detergent formulation.

Many works have reported the usefulness of alkaline proteases in the improvement of blood stain removal from cotton cloth at 40 °C [33, 71, 72]. Increasing time wash and temperature did not enhance blood removal, advising the usefulness of the crude extract in industrial applications as a cleaning bio-additive in detergent formulation at 20 °C reducing the energy input usually required in this type of application.

## Conclusion

We present in this study a cold-adapted bacterium with the ability of degrading feathers and producing a cold-active protease with keratinolytic activity. Only few bacteria have been reported to have this activity at cold-active temperatures. To our knowledge, this is the first report of a *Pedobacter* species being able to do this. It was proved that *Pedobacter* sp. 3.14.7 expresses only one protease/keratinase, which was extensively characterized. This enzyme exhibited high catalytic activity at moderate or low temperatures and proved to be resistant to harsh conditions. An excellent stability and compatibility with locally available commercial detergents at various temperatures has been established.

The results of the wash performance analysis demonstrated considerably good de-staining at room temperature and at 50°C for 40 min, indicating that *Pedobacter* proteolytic extract can be considered as a good detergent-additive in detergent industry.

Degradation of poultry waste with the concomitant production of a cold-active keratinase by this cold-tolerant microorganism might be attractive as an alternative for reducing the energy input required for biotechnological processes and their industrial applications.

## Data Availability

All data generated or analyzed during this study are included in this published article.

## Abbreviations

DMSO: Dimethyl sulfoxide
Ea: Activation energy for thermal inactivation of partially purified protease
EDTA: Ethylenediaminetetraacetic acid
FMM: Feather liquid mineral media
NA: Nutrient Agar plates
PMSF: Phenylmethylsulphonyl fluoride
PPP: Partially purified protease
R: Universal gas constant [8.314 J/(mol K)]
SDS: Sodium dodecyl sulfate
SDS-PAGE: SDS-polyacrylamide gel electrophoresis
*t*: Time
*T*: Temperature
TCA: Trichloroacetic acid
U: Unit of protease activity
